# Generation of equatorial plasma bubble after the 2022 Tonga volcanic eruption

**DOI:** 10.1038/s41598-023-33603-3

**Published:** 2023-05-22

**Authors:** Atsuki Shinbori, Takuya Sori, Yuichi Otsuka, Michi Nishioka, Septi Perwitasari, Takuo Tsuda, Atsushi Kumamoto, Fuminori Tsuchiya, Shoya Matsuda, Yoshiya Kasahara, Ayako Matsuoka, Satoko Nakamura, Yoshizumi Miyoshi, Iku Shinohara

**Affiliations:** 1 Institute for Space-Earth Environmental Research, Nagoya University, Chikusa-ku, Nagoya, 464-8601 Japan; 2 National Institute of Information and Communications Technology, Koganei, Tokyo 184-8795 Japan; 3 The University of Electro-Communications, Chofu, Tokyo 182-8585 Japan; 4 Department of Geophysics, Tohoku University, Aoba-ku, Sendai, 980-8578 Japan; 5 Planetary Plasma and Atmospheric Research Center, Tohoku University, Aoba-ku, Sendai, 980-8578 Japan; 6 Graduate School of Natural Science and Technology, Kanazawa University, Kakuma-machi, Kanazawa, 920-1192 Japan; 7 Word Data Center for Geomagnetism, Graduate School of Science, Kyoto University, Sakyo-ku, Kyoto, 606-8502 Japan; 8 Institute of Space and Astronautical Science, Japan Aerospace Exploration Agency, Chuou-ku, Sagamihara, 252-5210 Japan

**Keywords:** Natural hazards, Space physics

## Abstract

Equatorial plasma bubbles are a phenomenon of plasma density depletion with small-scale density irregularities, normally observed in the equatorial ionosphere. This phenomenon, which impacts satellite-based communications, was observed in the Asia-Pacific region after the largest-on-record January 15, 2022 eruption of the Tonga volcano. We used satellite and ground-based ionospheric observations to demonstrate that an air pressure wave triggered by the Tonga volcanic eruption could cause the emergence of an equatorial plasma bubble. The most prominent observation result shows a sudden increase of electron density and height of the ionosphere several ten minutes to hours before the initial arrival of the air pressure wave in the lower atmosphere. The propagation speed of ionospheric electron density variations was ~ 480–540 m/s, whose speed was higher than that of a Lamb wave (~315 m/s) in the troposphere. The electron density variations started larger in the Northern Hemisphere than in the Southern Hemisphere. The fast response of the ionosphere could be caused by an instantaneous transmission of the electric field to the magnetic conjugate ionosphere along the magnetic field lines. After the ionospheric perturbations, electron density depletion appeared in the equatorial and low-latitude ionosphere and extended at least up to ±25° in geomagnetic latitude.

## Introduction

Earth’s ionosphere, the partially ionized region of the upper atmosphere from 80 to 1000 km by solar extreme ultraviolet radiation^1^, includes the mesosphere and thermosphere. A height profile of plasma density in the ionosphere shows a peak of around 300 km, with the region of the highest plasma density above 150 km being called the F-region. The ionosphere below the F-region is called the E-region. A phenomenon known as the equatorial plasma bubble (hereafter, EPB) manifests as a plasma density depletion in the nighttime F-region of the equatorial ionosphere by exhibiting small-scale density irregularities inside the depressed region^2–5^. Geographically, an EPB extends from the equator to higher latitudes, with magnetic conjugacy^6–10^ being generated at the bottom side of the F-region at the equator by the nonlinear evolution of the Rayleigh-Taylor instability and rapid uplifting of the F-region^11^. As plasma density irregularity is the major driver of the refraction and scattering of radio waves, the EPB is arguably one of the key space weather phenomena nowadays^12–14^. Therefore, it is essential to identify the seed perturbation in plasma density triggered by acoustic and gravity waves, stemming from lower atmospheric phenomena.

Volcanic eruptions, earthquakes, tsunamis, and weather phenomena all trigger ionospheric disturbances that can propagate thousands of kilometers from the origin^15–22^. Recently, the Hunga Tonga-Hunga Ha’apai (hereafter, Tonga) volcanic eruption occurred at 04:15 UTC on January 15, 2022, and generated multiple air pressure waves, which were subsequently detected by ground weather stations around the world^23^. These atmospheric perturbations propagated upward as acoustic and gravity waves and became the main driver of ionospheric perturbations^24–27^. In turn, the ionospheric disturbances consisted of concentric traveling ionospheric disturbances that propagated outward from the eruption point. The propagation speed corresponded to the acoustic speed around the peak altitude of the F-region. Thus, the Tonga volcanic eruption generated ionospheric disturbances that could have become a seed for the generation of an EPB^27–30^. The previous study found the EPB occurrence in the Asia-Pacific region after the Tonga volcanic eruption using Swarm satellite in-situ plasma data and discussed several physical mechanisms of the generation of EPB: direct seeding effect, destabilization of the bottom side of the F-region, and upward motion of the ionosphere after sunset, leading to an enhancement of Rayleigh-Taylor instability in the F-region^28^. The EPB observed over 100–150°E extended to higher latitudes (~40°N in geographic latitude) with a magnetic conjugate feature due to the strong pre-reversal enhancement in the dusk sector^29^. The ionospheric radar observed the F-region irregularity related to the EPB occurrence over India^30^.

Our motivation for this study is that the Geospace satellite Arase^31^ for the first time observed the electron density depletion associated with an EPB at the height of the F-region just after air pressure waves passed into the troposphere. Although there are some reports on the EPB occurrence after the Tonga volcanic eruption^27–30^, they could not clarify that air pressure waves generated the EPB and related ionospheric disturbances, based on direct observations because they used the anticipated results regarding a Lomb wave propagation. Therefore, we had a unique opportunity in that the Arase satellite detected the EPB occurrence during an evening sector around the initial arrival of air pressure waves captured by the Himawari-8 satellite. In this study, we demonstrate that the EPB was generated by air pressure waves due to the largest-on-record January 15, 2022 eruption of the Tonga volcano by analyzing satellite and ground-based observations. Finally, we identify the plasma density perturbation and ionospheric height increase becoming a cause of the EPB occurrence and investigate the relationship between these ionospheric disturbances and air pressure waves in the troposphere.

## Observation of the equatorial plasma bubble after the Tonga volcanic eruption

For achieving our objectives, we used the global navigation satellite system (GNSS)-total electron content (TEC) observation data. To identify the occurrence of ionospheric small-scale plasma density irregularities, we calculated the rate of TEC index (ROTI), defined as a standard deviation of the time derivative of TEC (rate of TEC changes, or ROT) for each 5-min time interval^32^. Note that ROTI values have often been utilized as an efficient indicator of the occurrence of an EPB^33^. Details of how to derive the TEC, ROT, and ROTI are described in section "Derivation of TEC, ROT, and ROTI grid data from RINEX (Receiver Independent Exchange Format) files". We also analyzed ionosonde data to investigate a vertical motion of the F-region which is one of the important elements for EPB generation. We also examined ionospheric electron density variations, estimated from the in-situ plasma waves, observed by the high frequency analyzer^34^, a subsystem of the plasma wave experiment^35^ onboard the Arase satellite. Derivation of the electron density from the in-situ plasma waves is described in section "Estimation of electron density in the inner magnetosphere and ionosphere from Arase plasma wave observations". Further, we analyzed thermal infrared (TIR) data of 6.2 μm wavelength, acquired from Himawari-8 satellite measurements^36,37^ to elucidate the signature of air pressure waves in the troposphere. Identification of air pressure waves from the TIR data is explained in section "Calculation of temperature deviation of the TIR grid data obtained from the Himawari 8 satellite".

**Figure 1:**
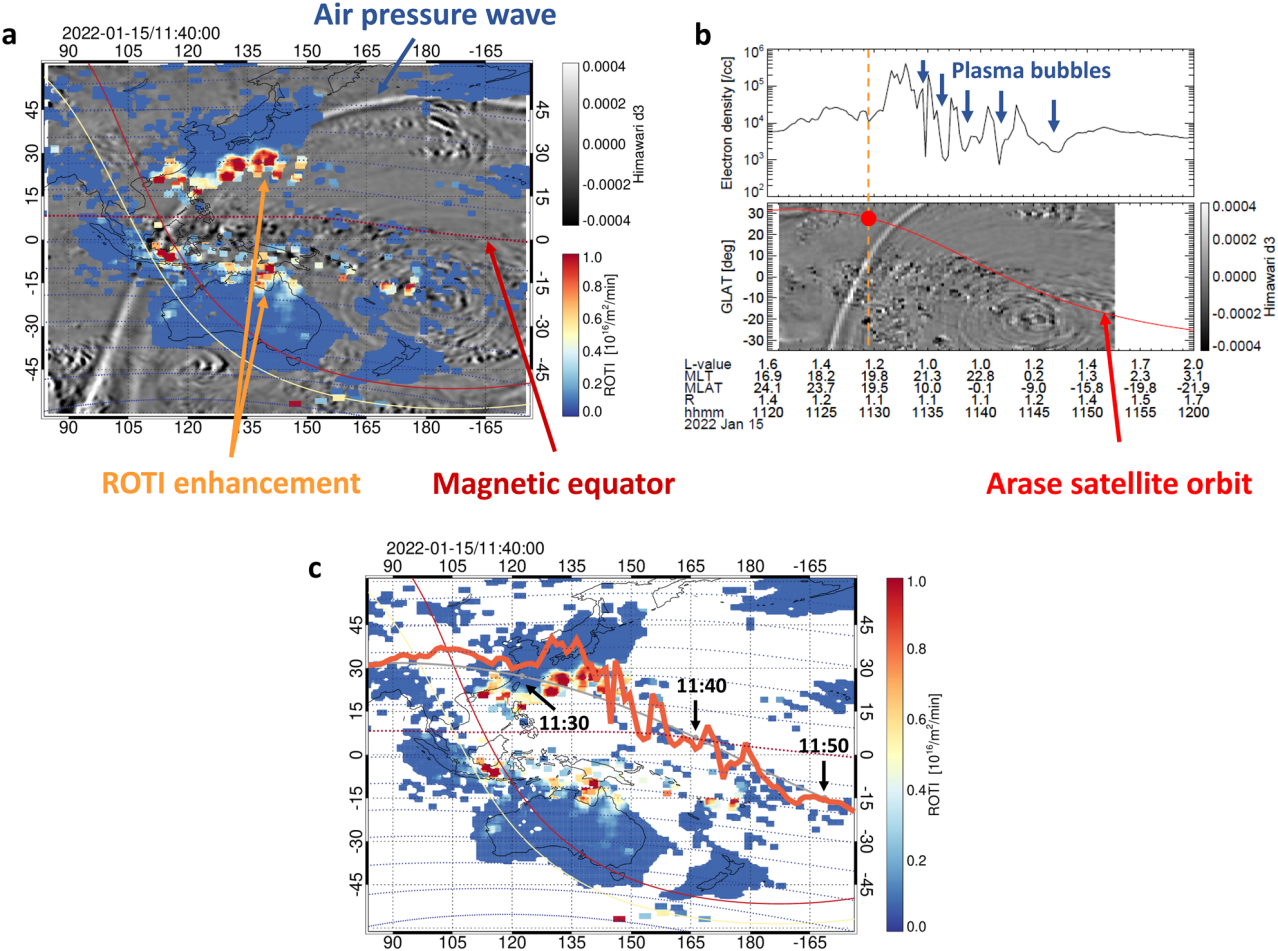
Observation of the equatorial plasma bubble (EPB) and air pressure wave between 11:20 to 12:00 UTC on January 15, 2022. (**a**) Two-dimensional map of ROTI values and the Himawari-8 temperature deviation (d3) at 11:40 UT, indicated by the color and gray scales, respectively. The yellow and red lines indicate the sunset at 105 and 300 km heights. The horizontal dashed curves represent the geomagnetic latitude every 10°. (**b**) Electron density observed by the Arase satellite and geographic latitude-time plot of Himawari-8 d3 data along the geographic longitude of the Arase satellite position. (**c**) Two-dimensional map of ROTI values with the electron density along the Arase satellite orbit in the same period as in Fig. 1b. The smoothed gray curve indicates the Arase satellite orbit, while the thick orange line around the smoothed curve indicates the electron density variation.

A two-dimensional map of ROTI values and the deviation of TIR temperature in the Asia-Pacific region, shown in Fig. 1a, reveals a significant enhancement of ROTI values of more than 0.4 in the low-latitude regions of at least ±25° in magnetic latitude before the initial arrival of air pressure waves in the troposphere. Although the sensitivity of the ROTI signature tends to increase with an increase in the ionospheric electron density associated with the equatorial ionization anomaly, the ROTI enhancement suggests the existence of ionospheric plasma density irregularity^33,38^. In this figure, the wavefront of the air pressure waves can be seen as a white arc with a value of more than 0.0003 indicated by the blue arrow. Furthermore, the ROTI enhancement began emerging after the sunset of the E- or F-region of the ionosphere. Movie S1 (Supplementary Information) clearly illustrates the westward extension of the enhanced ROTI region, which corresponds to the propagation of air pressure waves. Moreover, an electron density depletion with values of one order or more was identified after a sudden increase of electron density with more than one order magnitude at 11:29:29 UTC by Arase observations (Fig. 1b). At that time, the satellite was traveling at the altitude of sunset for the F-region from the Northern to Southern Hemispheres. The altitude of the electron density depletion ranged from 409 to 2032 km, corresponding to the F-region of the ionosphere to the lower plasmasphere. Although such electron or ion density depletion after the Tonga volcanic eruption was already found by several low earth orbit satellites at the altitude range from 450 to 575 km^28^, the Arase in-situ observation showed that the region of electron density depletion extended to higher altitudes of at least 2000 km. Because the previous study used low earth orbit satellite data^28^, it could not detect plasma density depletion at higher altitudes. Referring to the magnitude of the electron density depletion observed by previous satellite observations^39^, the phenomenon corresponds to an EPB. The electron density depletions, based on the Arase observations, generally exhibited a good overlap with the ROTI enhancement area in the Asia-Pacific region (Fig. 1c). These findings indicate that the ROTI enhancement corresponded to the occurrence of an EPB and that the EPB generation was strongly associated with the neutral atmospheric disturbances propagating in the lower atmosphere, which had been triggered by the Tonga volcanic eruption. Further, the EPB extended to higher altitudes where the footprint of the magnetic field line corresponds to ~30° in geomagnetic latitude. This result supports that reported by the previous study^27^.

## Comparing ionospheric motion and air pressure waves in the troposphere

**Figure 2:**
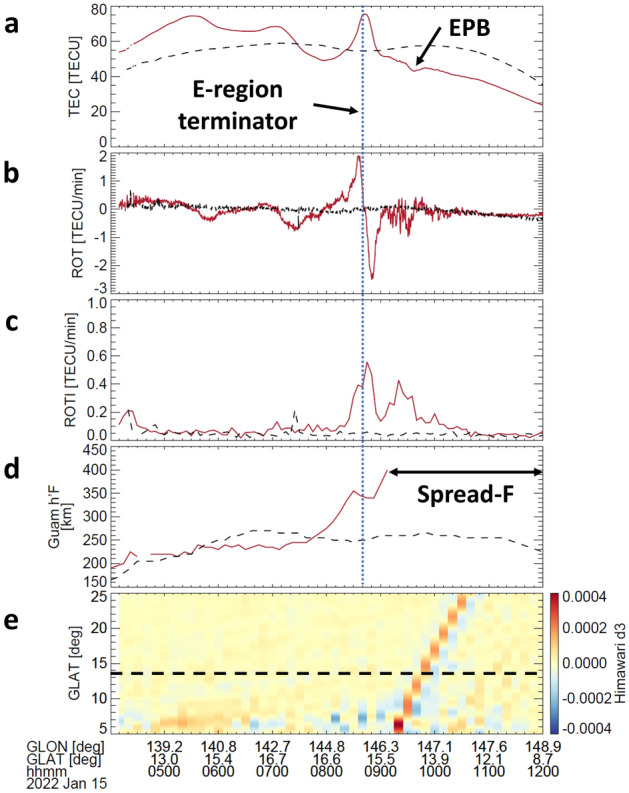
Comparison between the TEC, ionosonde, and Himawari-8 satellite observations. (**a**–**c**) Time-series plots of TEC, ROT, and ROTI at GUUG obtained from PRN16 (corresponding to the GPS satellite number). (**d**) Time-series plots of h’F at a frequency of 4 MHz observed by the ionosonde in Guam. The black dashed lines in each panel (**a**–**d**) indicate the values of each parameter during 04:00–12:00 UTC on January 13, 2022. (**e**) Geographic latitude-time plot of Himawari-8 temperature deviation (d3). The horizontal dashed line represents the location of the ionosonde at the Guam station. The geographic longitude and latitude shown in the bottom panel (**e**) indicate the ionospheric pierce point at a height of 300 km between the GUUG station and PRN16.

**Figure 3:**
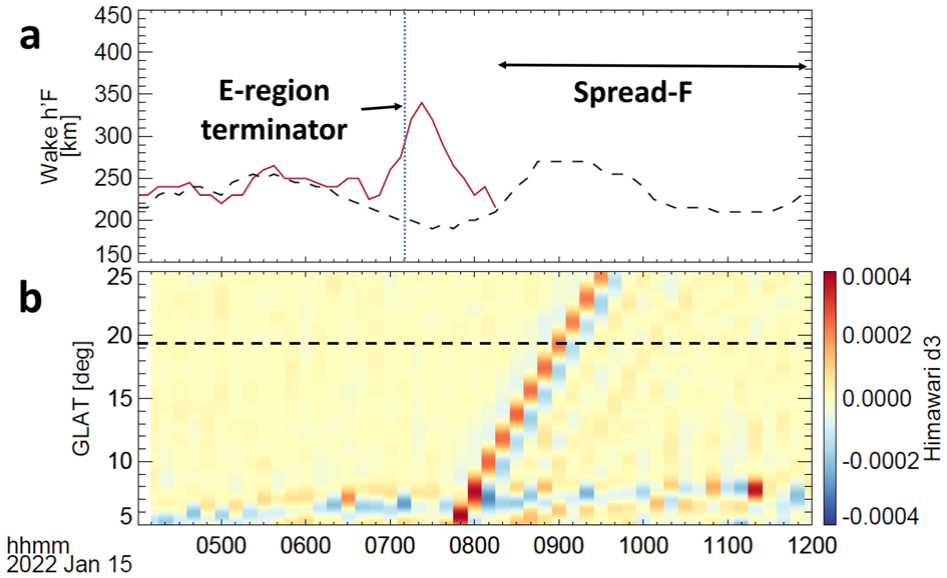
Comparison between the ionosonde and Himawari-8 satellite observations. (**a**) Time-series plot of h’F variation at a frequency of 4 MHz at Wake. (**b**) Geographic latitude time plot of temperature deviation (d3) aligned with the geographic longitude of 166.64°E observed by the Himawari 8 satellite during 04:00–12:00 UTC on January 15, 2022. The vertical dotted line in panel (**a**) reflects the sunset terminator of the E region height of 105 km. The horizontal dashed line in panel (**b**) is the geographic latitude of the Wake station.

The sudden increase of electron density before the arrival of air pressure waves (Fig. 1b) suggests the uplift of the F-region to a higher altitude. This motion induced an enhancement of the growth rate of the Rayleigh-Taylor instability that produces an EPB. Fig. 2 shows a relationship between the variations of the observed TEC at the GUUG station (13.43°N, 144.80°E) and the height of the F-region (hereafter, h’F) at the Guam station (13.62°N, 144.86°E). The spatial relation between the GUUG and Guam stations is 23.17 km (300 km altitude) away in the north-northeast direction. The h’F value exhibited a rapid increase from 245 to 400 km after 07:37 UTC near the terminator of the E-region (Fig. 2d), thereby indicating the increasing height of the F-region. After 09:07 UTC, the h’F value could not be interpreted from ionograms due to diffused F-region echoes (spread-F) (Fig. S1). In Fig. 2c and d, the h’F increases at Guam station are temporally well-correlated with corresponding ROTI increases at GUUG station. Fig. 2c also shows two sudden increases in ROTI after h’F increases. The first ROTI enhancement corresponded to a sudden increase and decrease of TEC with large ROT, and the second enhancement was triggered by the TEC perturbations, related to an EPB with small-scale plasma density irregularity (Fig. 2a and b) after the E-region became dark. A slight depletion of TEC associated with EPB was identified around 09:35 UT, as seen in Fig. 2a. Furthermore, a comparison between the ionospheric and atmospheric observations revealed that the upward motion of the ionosphere occurred ~2 hours before the arrival of air pressure waves in the troposphere at the Guam station, as shown in Fig. 2e, although the previous study reported that the upward motion was observed around the anticipated arrival time of a Lamb wave^28^. At the Wake station (19.294°N, 166.647°E) located at ~20° longitude from the Guam station, the h’F value suddenly increased and decreased (Fig. 3) after 06:45 UTC. The magnitude of the h’F variation was 115 km. The spread of F critically hampered the acquisition of the h’F value from ionograms after 07:15 UTC (Fig. S2). It is noted that the h’F value is also changed by the upward motion of the ionosphere and other processes such as an enhancement of recombination at lower heights. In this case, the EPB occurred around the sunset terminator of the E- and F-regions after an increase of h’F. Therefore, the EPB occurrence suggests that the upward motions of the ionosphere near the sunset terminator of the E-region ensured conducive conditions^3–5,40^. Recently, the high-resolution atmosphere-ionosphere coupled model revealed an upward plasma motion transporting low-density plasma in the bottom side of the F-region to higher altitudes associated with atmospheric disturbances triggered by the Tonga eruption^41^.

**Figure 4:**
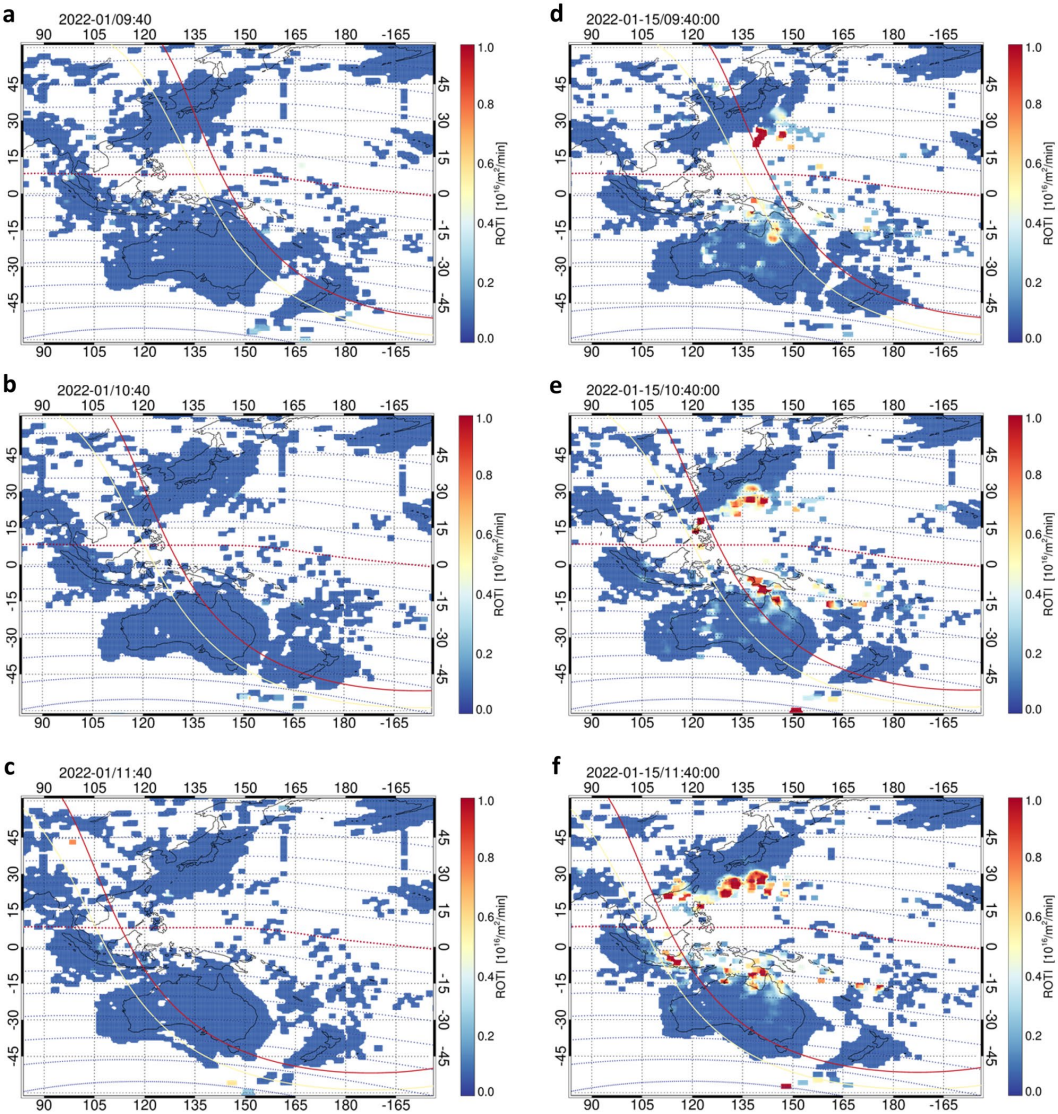
Comparison between the average ROTI in January 2022 and the ROTI on the day of the Tonga eruption (15 January 2022). (**a**–**c**) Two-dimensional maps of the average ROTI at 09:40, 10:40, and 11:40 UTC, respectively, in January except for the eruption day. (**d**–**f**) Two-dimensional maps of the ROTI at the same time in panels (**a**–**c**) on the eruption day. The yellow and red lines indicate the sunset at 105 and 300 km heights. The horizontal dashed curves represent the geomagnetic latitude every 10°.

It is important to elucidate a difference in the occurrence features of EPBs between the normal and eruption days to evaluate the seeding effect of atmospheric disturbances triggered by the volcanic eruption on EPBs. Figure 4 shows a comparison between the two-dimensional maps of the average ROTI in January and the ROTI on the eruption day. In Fig. 4a–c, the ROTI enhancement indicating the occurrence of EPBs cannot be seen clearly after the sunset terminators at an altitude of the E- and F-regions. The low activity of EPBs in the Asia-Pacific region in January is consistent with the EPB occurrence shown by several statistical analysis results of satellite observations^42–45^. However, in Fig. 4d–f, the ROTI enhancement appears in the equatorial and low-latitude regions after the sunset terminators at an altitude of the E- and F-regions. The high activity of EPBs on Tonga eruption day is quite different from the normal activity of EPBs in the Asia-Pacific region in January. This result suggests that the abnormal occurrence of EPBs is mainly caused by the seeding effect of atmospheric pressure waves triggered by the Tonga volcanic eruption.

**Figure 5:**
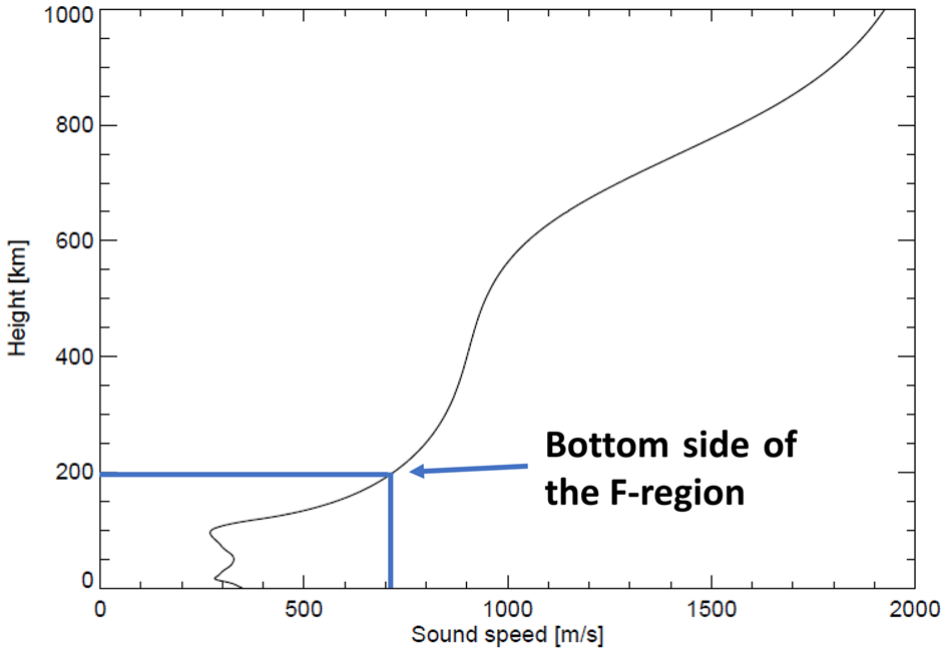
Height profile of sound speed from the troposphere to the thermosphere (0–1000 km). The calculation date and time are at 04:00 UTC on January 15, 2022. The geographic latitude and longitude of the calculation point are 0° and 135°E, respectively.

Moreover, in Figs. 2 and 3, the upward motion of the ionosphere began ~52 min before at the Wake station than at the Guam station. Given the shorter distance between the Tonga volcano and the Wake station (4848.7 km), compared to that between the Tonga volcano and the Guam station (5778.1 km), a difference in the onset times reflects the time when acoustic waves reached each station from the Tonga volcano. How to obtain these distances from the Tonga volcano and each ionosonde station is described in section "Calculation method of the distance between each observation point and the Tonga volcano". By assuming that the atmospheric disturbances occurred exactly over the Tonga volcano, the propagation speed was estimated to be ~476.7 m/s (Guam-Tonga) and 538.7 m/s (Wake-Tonga). Thus, the propagation speed was higher than that of a Lamb wave (~315 m/s) propagating in the troposphere. These findings indicate that the acoustic waves that had triggered the upward and downward motions of the ionosphere propagated in the thermosphere. As shown in Fig. 5, the speed of the acoustic waves (~700 m/s at an altitude of 200 km corresponding to the bottom side of the F-region) was markedly higher compared with that in the troposphere (~315 m/s). The calculation method of the acoustic waves is shown in section "Calculation of speed of sound in the neutral atmosphere from the troposphere to the thermosphere". In addition to the observational evidence that the propagation speed of the ionospheric disturbances was higher than that of a Lamb wave, the present study showed that the propagation speed tended to decrease with an increase in the distance from the Tonga volcano and each ionosonde station. The previous study found that the propagation speed of the ionospheric disturbances had a wide range from ~1050 to 315 m/s around the Tonga volcano and that the fast wave (~1050 m/s) did not propagate beyond 1000 km^28^. This result suggests that the slow wave can propagate in the ionosphere over a long distance as reported by another study^24^. Therefore, the propagation speed of the ionospheric disturbances is slowed down with an increase in the distance. This is the main reason why the propagation speed obtained by the present study is different from that reported by previous studies^24,28^.

## Spatial distribution of time difference between ionospheric and atmospheric response

**Figure 6:**
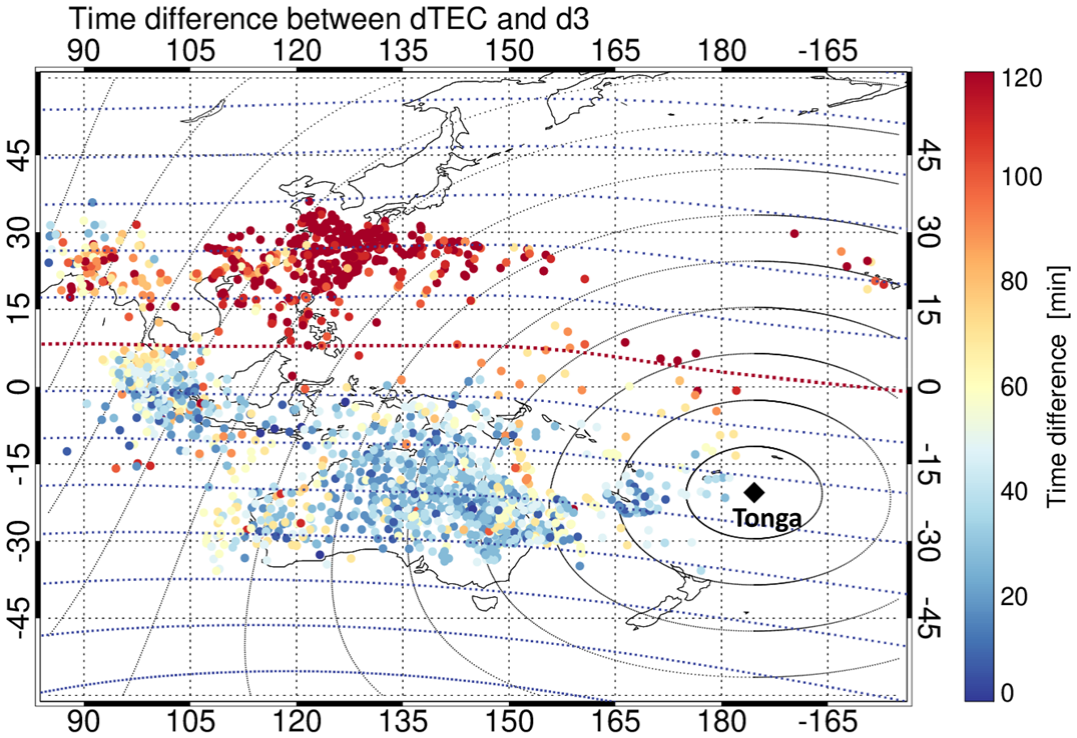
Spatial distribution of the time difference between the onset times of TEC perturbation in the ionosphere and temperature deviation in the troposphere. The horizontal and vertical axes indicate the geographic longitude and latitude in degrees, respectively. The color scale indicates the time difference ranging from 0 to 120 min. The red dotted curve is the magnetic equator. The concentric circles represent a distance from the Tonga volcano every 1000 km.

Figures 2 and 3 show that the upward motion of the ionosphere that requires EPB generation occurred before the initial arrival of the air pressure waves and propagated in the troposphere as a Lamb mode, thereby demonstrating that the acoustic waves generated by the Tonga volcanic eruption could propagate in the thermosphere. We hypothesized that because the speed of acoustic waves is higher in the thermosphere than in the troposphere, the time difference between the onset periods of the ionospheric perturbation and temperature deviation in the troposphere, triggered by the air pressure wave, increased with increasing distance from the Tonga volcano to the observation point. To evaluate this hypothesis, we analyzed the spatial distribution of the time difference between the onset times of TEC perturbation and temperature deviation in the Asia-Pacific region. To this end, we opted for the TEC data, obtained from 264 GNSS stations located within ±30˚ in geographic latitude, as shown in Fig. 6. Most importantly, Fig. 6 shows that the TEC perturbations preceded the tropospheric temperature deviation for all the events, while the time difference did not necessarily increase with increasing distance from the Tonga volcano. This finding indicates that the atmospheric disturbances propagating in the thermosphere as an acoustic wave were not generated only over the Tonga volcano but also on the way between each observation point and the volcano. Moreover, the onset of the TEC perturbations before the initial arrival of the atmospheric disturbances can be explained by the forward energy leakage of the acoustic waves that propagated with a markedly higher speed in the thermosphere than in the troposphere^46^.

At the same time, the spatial distribution of the time difference from Fig. 6 illustrates that the points with > 100 min were clustered in the Northern Hemisphere around Japan. The considerably fast response of the ionosphere to the arrival of atmospheric disturbances cannot be fully explained by the forward energy leakage of the acoustic waves in the thermosphere. In this context, recent studies on traveling ionospheric disturbances just after the Tonga volcanic eruption have reported that the ionospheric disturbances appeared in the Northern and Southern Hemispheres with magnetic conjugacy^47–50^. The generation mechanism of the ionospheric disturbances is somehow an instantaneous transmission of the electric field to the magnetic conjugate ionosphere along the magnetic field lines^49^. The electric field was generated by an E-region dynamo in the sunlit Southern Hemisphere, which was governed by the neutral wind oscillation in the thermosphere^49^. Overall, these findings demonstrate that the fast response of the ionosphere in the Northern Hemisphere around Japan can be attributed to the external electric field generated by the Southern Hemisphere.

## Summary and conclusions

To demonstrate the generation of EPBs in the equatorial and low-latitude ionosphere due to the seeding effect of air pressure waves triggered by the Tonga volcanic eruption, we conducted an integrated analysis of GNSS-TEC, ionosonde, Arase, and Himawari-8 satellite observations. As a result, the EPBs were observed in the Asia-Pacific region of the equatorial and low-latitude ionosphere (at least ±25° in geomagnetic latitude) several minutes to hours before the initial arrival of the air pressure waves propagating in the troposphere. The EPBs extended to a higher altitude of at least 2000 km, corresponding to the lower plasmasphere. The propagation speed of electron density perturbations in the ionosphere was in a range from ~480 to 540 m/s, whose speed was higher than that of a Lamb wave (~315 m/s) propagating in the troposphere. This result implies that the acoustic waves that trigger the upward and downward motions of the ionosphere propagate in the thermosphere. At the same time, the electron density perturbations tended to begin in the Northern Hemisphere around Japan more than 100 min before the initial arrival of the air pressure waves. The considerably fast response of the ionosphere could be caused by an instantaneous transmission of the electric field to the magnetic conjugate ionosphere along the magnetic field lines.

Various kinds of geophysical phenomena generated by the largest-on-record January 15, 2022, Tonga eruption have been previously recorded in a global dataset consisting of atmospheric waves^23,51^, tsunamis^52^, and ionospheric disturbances^24–30,47–50^, thereby providing a unique opportunity for an integrated study based on the synergy of multi-disciplinary observation data. This study provides clear evidence of the EPB appearance after the Tonga volcanic eruption, based on direct observations, thereby corroborating the existence of a seed effect of atmospheric disturbances, as shown by previous studies^27–30^. These results shed light on how to predict EPB occurrence.

### Supplementary Information

Supplementary Video S1.

Supplementary Information.

## Data Availability

The Receiver Independent Exchange Format data used for GNSS-TEC processing were provided by 50 data providers. These data have been listed on the webpage of the GNSS-TEC database (http://stdb2.isee.nagoya-u.ac.jp/GPS/GPS-TEC/gnss_provider_list.html). The main contributing providers were University Navistar Consortium (https://www.unavco.org/data/gps-gnss/gps-gnss.html), Crustal Dynamics Data Information System (https://cddis.nasa.gov/archive/gnss/data/daily/)^70^, Canadian high arctic ionospheric network (http://chain.physics.unb.ca/chain/pages/data_download)^71^, Pacific Northwest Geodetic Array (http://www.geodesy.cwu.edu)^72^, Instituto Brasileiro de Geografia e Estatística (http://geoftp.ibge.gov.br/informacoes_sobre_posicionamento_geodesico/rbmc/dados/), South Pacific Applied Geosciences Commission (http://garner.ucsd.edu), GNSS Earth Observation Network System (https://www.gsi.go.jp/ENGLISH/geonet_english.html), GNSS Earth Observation Network System (https://www.geonet.org.nz/data/types/geodetic), Reseau National GPS permanent (https://doi.org/10.15778/resif.rg)^73^, Systeme d'Observation du Niveau des Eaux Littorales (https://www.sonel.org/-GPS-.html), Land Information New Zealand (https://www.geodesy.linz.govt.nz/gdb/), Rete Integrata Nazionale GPS (http://ring.gm.ingv.it)^74^, Australian Space Weather Services (https://www.sws.bom.gov.au/World_Data_Centre/1/1), the African Geodetic Reference Frame (http://afrefdata.org/), Trans-boundary, Land and Atmosphere Long-term Observational and Collaborative Network (http://tlalocnet.udg.mx/tlalocnetgsac/), Northern California Earthquake Data Center (https://ncedc.org/bard.overview.html)^75^, Regional Reference Frame Sub-Commission for Europe (https://www.epncb.oma.be/)^76^, Red Argentina de Monitoreo Satelital Continuo (https://www.ign.gob.ar/NuestrasActividades/Geodesia/Ramsac/DescargaRinex)^77^, and the British Isles continuous GNSS Facility (http://www.bigf.ac.uk/data_access.html). The Himawari-8 satellite TIR grid data are provided by the Center for Environmental Remote Sensing, Chiba University (http://www.cr.chiba-u.jp/databases/GEO/H8_9/FD/index_en_V20190123.html). The present study used PWE/HFA L2 v01_01^78^, PWE/HFA L3 v01_02^79^, MGF-L2 v03_03^80^, OBT L2 v03^81^, and OBT L3 v02^82^ data.

## Estimation of electron density in the inner magnetosphere and ionosphere from Arase plasma wave observations

To investigate electron density variation in the topside ionosphere associated with an air pressure wave in the troposphere, we analyzed plasma wave observation data obtained from the Arase satellite^31^. The Arase satellite has an elliptical orbit with a perigee of 400 km altitude, an apogee of 32,110 km, an inclination of 31°, and an orbital period of 565 min. In this analysis, we first identified the upper limit frequency of the upper hybrid resonance (UHR) waves in the plasma wave dynamic spectra obtained from the Arase plasma wave experiment (PWE)-high frequency analyzer (HFA) instrument^33^. For determination of the upper limit frequency, we seek the narrow-banded spectra above the electron cyclotron frequency which vary as a function of the location of the Arase satellite and identify the frequency where the intensity of the narrow-banded spectra decreases abruptly up to the background level. The upper limit frequency ${f}_{UHR}$ is expressed using Eq. (1):

1 $${f}_{UHR}= \sqrt{{f}_{pe}^{2}+{f}_{ce}^{2},}$$

where the parameters ${f}_{pe}$ and ${f}_{ce}$ are electron plasma and cyclotron frequencies, respectively. These frequencies are expressed using Eqs. (2) and (3), respectively:

2 $${f}_{pe}=\frac{1}{2\pi }\sqrt{\frac{{e}^{2}n}{{\varepsilon }_{0}{m}_{e}}},$$

3 $${f}_{ce}= \frac{1}{2\pi }\frac{eB}{{m}_{e}},$$

where the parameters $e$, $n$, ${m}_{e}$, ${\varepsilon }_{0}$, and $B$ are electron charge, density, mass, permittivity in a vacuum, and magnetic field intensity, respectively. Therefore, if we know the upper limit frequency of the UHR waves, we can easily derive the electron density from Eqs. (1), (2) and (3). The time resolution of the electron density data fundamentally depends on the operation mode of the PWE-HFA instrument. In this calculation, we used the magnetic field intensity obtained from the magnetic field instrument^53^ onboard the Arase satellite. These plasma wave and magnetic field data of the Exploration of energization and Radiation in Geospace (ERG) (Arase) satellite were obtained from the ERG Science Center^54^ operated by the Institute of Space and Astronautical Science, Japan Aerospace Exploration Agency and Institute for Space-Earth Environmental Research, Nagoya University (https://ergsc.isee.nagoya-u.ac.jp/index.shtml.en).

Fig. S3 shows an example of plasma wave dynamic spectra and electron density in the topside ionosphere during 11:20–12:00 UTC on January 15, 2022, observed by the Arase satellite. The narrow-banded spectra below the red line are the UHR and Z-mode waves, whose observed frequencies depend on the electron density and ambient magnetic field intensity. In this case, the temporal variation of the upper limit frequency of the UHR waves almost reflects the electron density perturbation along the Arase satellite orbit.

## Calculation of temperature deviation of the TIR grid data obtained from the Himawari 8 satellite

We noted that the atmospheric disturbances due to Lamb waves triggered by the Tonga volcanic eruption are markedly faster than cloud motions in the troposphere and calculated the normalized deviation from the simple running average of the thermal infrared (TIR) grid data. The spatial resolution of the grid data is 0.02˚×0.02˚ in geographical latitude and longitude. The normalized deviation was calculated using Eqs. (4) and (5):

4 $${T}_{avg}\left(t\right)=\frac{{T}_{bb}\left(t+10\right)+{T}_{bb}\left(t\right)+{T}_{bb}\left(t-10\right)}{3},$$

5 $${d}_{3}\left(t\right)=\frac{{{T}_{bb}\left(t\right)-T}_{avg}\left(t\right)}{{T}_{avg}\left(t\right)},$$

where ${T}_{bb}$, $t$, ${T}_{avg}$, and ${d}_{3}$ indicate the thermal infrared temperature (K), time (min), simple running average, and normalized deviation from simple running average of the TIR gird data, respectively. In this study, we used the normalized temperature deviation (${d}_{3}$) of ${T}_{bb}$ in a comparison with GNSS-TEC observation data. Furthermore, we conducted a 50×50 smoothing for the normalized temperature deviation data obtained from Eqs. (4) and (5) to efficiently identify the wavefront of the air pressure wave propagating as a Lamb mode just after the Tonga volcanic eruption.

## Derivation of TEC, ROT, and ROTI grid data from RINEX (Receiver Independent Exchange Format) files

We derived the GNSS-TEC value with a time resolution of 30 s from the collected GNSS data according to the procedure described in several papers^55–58^. To quantify the range between the satellite and receiver, we need to know the time delay of the pseudorandom-noise code sequences of the received signal. However, this value includes several errors such as tropospheric and ionospheric effects. The pseudorange *p* is written as:

6 $$p=\rho +c\left(dt-dT\right)+{d}_{I}+{d}_{T}+dq+dQ+{\epsilon }_{p},$$

where $\rho$ is the geometric range between satellite and receiver, $c$ is the speed of light in a vacuum, $dt$ is the offset of satellite clock, $dT$ is the offset of receiver clock, ${d}_{I}$ is the ionospheric delay, ${d}_{T}$ is the tropospheric delay, $dq$ is the instrumental bias of the satellite, $dQ$ is the instrumental bias of the receiver, and ${\epsilon }_{p}$ is the random error on pseud-oranges. Based on the refractive index of an electromagnetic wave propagating in a plasma, the ionospheric delay ${d}_{I}$ becomes

7 $${d}_{I}=\frac{K}{{f}^{2}}\int {n}_{e}ds,$$

where ${n}_{e}$ is the electron density along the ray path $s$ and $K=40.3$ m^3^s^-1^.

As ${d}_{I}$ depends on the frequency of electromagnetic waves, pseudorange differences at the two different frequencies cancel out the unknown range $\rho$, the clock offsets, and the tropospheric delay in eq. (7), and provide a TEC expression $I=\int {n}_{e}ds$. Because the differential instrumental biases do not vanish, the pseudorange TEC value is noisy and low accuracy. In a similar manner, we can also derive a TEC value from the carrier phase differences at the two different frequencies. This value stands out with high accuracy but includes uncertainty in an absolute scale by multiples of wavelength. To solve this problem, we adjusted the carrier phase TEC value to the pseudorange value.

As the calculated relative TEC value contained the instrumental biases, the determination of the absolute TEC value required the accurate estimation of a bias. To this end, we applied the method proposed by the previous study^59^. This method estimates 1-h average TEC and inter-frequency biases by weighted least squares fitting of the relative TEC value for each GNSS station and excludes the biases from the relative TEC values. A detailed description of the estimation of the absolute TEC value can be found in the following study^59^. Furthermore, we calculated the vertical TEC value from the slant value by multiplying a slant factor ($1/S\left(\epsilon \right)$), which is a ratio of the ionospheric thickness. Here, S and $\epsilon$ are the slant factor and elevation angle of the GNSS satellite, respectively^60^. The ionospheric thickness varies from 200 to 600 km, depending on latitude, longitude, local time, season, and solar activity^61^. However, because we cannot know the direct value of the global ionospheric thickness, we assume that the ionospheric thickness is a constant value of 200 km within a height range from 250 to 450 km as a zero-order approximation.

Assuming that ionospheric plasma density irregularities do not change within a short time while they pass a line of site between satellite and receiver, the rate of TEC changes (ROT) obtained from the time derivative of TEC corresponds to spatial gradients of TEC. To determine the small-scale irregularities statistically, we defined a rate of TEC index (ROTI) as a standard deviation of ROT for 5 min. The ROTI expression ${R}_{I}$ is written in the following equation using the ROT (${R}_{T}$)

8 $${R}_{I}=\sqrt{\langle {R}_{T}^{2}\rangle -{\langle {R}_{T}\rangle }^{2}},$$

9 $${R}_{T}\left({t}_{m}\right)=\frac{{I}_{m}-{I}_{m-1}}{{t}_{m}-{t}_{m-1}},$$

where ${t}_{m}$ is the time in seconds at the data number $m$, and $I$ is the TEC. Because the ROTI is a standard deviation of ROT for 5 min, the time interval of the ROTI data is 5 min.

Finally, we mapped the vertical TEC and ROTI values onto the thin shell ionosphere at an altitude of 300 km. In this method, we set the maximum satellite zenith angle to 75°, while composing the grid data of the TEC and ROTI. The spatial resolution of these grid data was 0.5°×0.5° in longitude and latitude. For the TEC and ROTI data, we also conducted a pixel smoothing with a boxcar window of 2.5°×2.5° in geographic latitude and longitude every 5 min and converted them into netCDF-formatted data to efficiently interpret the TEC and ROTI data using Space Physics Environment Data Analysis System^62^ and Inter-university Upper atmosphere Observation NETwork (IUGONET)^63–66^ data analysis software (UDAS)^67^. These GNSS-TEC data are stored in a database (http://stdb2.isee.nagoya-u.ac.jp/GPS/GPS-TEC/) managed by the PWING (study of dynamical variation of Particles and Waves in the INner magnetosphere using Ground-based network observations) project^68^.

## Calculation method of the distance between each observation point and the Tonga volcano

This calculation assumed that the earth is a sphere with an equatorial radius of 6378.137 km. The distance $d$ between the observation point A(${x}_{1},{y}_{1}$) and the location of the Tonga volcano B(${x}_{2},{y}_{2}$) is written as the following expression:

$$d=r{cos}^{-1}\left(sin{y}_{1}sin{y}_{2}+cos{y}_{1}cos{y}_{2}cos\Delta x\right)$$

10 $$\phi =90-atan\left(\frac{sin\Delta x}{cos{y}_{1}tan{y}_{2}-sin{y}_{1}cos\Delta x} \right),$$

$$\Delta x={x}_{2}-{x}_{1},$$

where ${x}_{1},$ ${y}_{1},$ ${x}_{2},$ ${y}_{2}$ are the geographic latitude and longitude of the points A and B, $r$ is the equatorial radius of the earth, and $\phi$ is the azimuth angle in degrees.

## Calculation of speed of sound in the neutral atmosphere from the troposphere to the thermosphere

The speed of sound $c$ is expressed using the Newton–Laplace equation:

11 $$c=\sqrt{\frac{K}{\rho }},$$

where $K$ and $\rho$ are the modulus of bulk elasticity for gases and the density, respectively. For an ideal gas, $K$ is expressed as:

12 $$K=\gamma p.$$

Thus, the speed of sound in eq. (11) is replaced by

13 $$c=\sqrt{\frac{\gamma p}{\rho }},$$

where $\gamma$ and $p$ are the adiabatic index and pressure, respectively. The adiabatic index is defined as the ratio of the specific heat of a gas at constant pressure to that of a gas at constant volume (${C}_{p}/{C}_{v}$). Using the ideal gas law,$p$ and $\rho$ are replaced with $nRT/V$ and $nM/V$, respectively. Here, $n$, $R$, $T$, $V$, and $M$ represent the number of moles, gas constant (8.314 [J/K/mol]), temperature, volume, and the mass of the gas, respectively. Finally, the equation for an ideal gas becomes

14 $$c=\sqrt{\frac{\gamma RT}{M}}.$$

Because Earth’s atmosphere is composed of several atoms and molecules, and the composition changes as a function of height, we calculated the average adiabatic index $\overline{\gamma }$ and mass of the gas $\overline{M }$ using the following equations to derive the speed of sound:

15 $$\overline{\gamma }=\frac{\sum_{i}{\gamma }_{i}{n}_{i}}{\sum_{i}{n}_{i}},$$

16 $$\overline{M }=\frac{\sum_{i}{M}_{i}{n}_{i}}{\sum_{i}{n}_{i}},$$

where ${\gamma }_{i}$, ${n}_{i}$, and ${M}_{i}$ are the adiabatic index, number density, and mass of each component of the atmosphere, respectively. ${\gamma }_{i}$ is exactly 5/3 = 1.667 for monoatoms (such as Ar), 7/5 = 1.4 for diatomic molecules (such as N_2_) and 4/3 = 1.333 for triatomic molecules like CO_2_. The parameters required to calculate the speed of sound are obtained from the NRLMSIS00 model^69^. Based on the above calculation method, the speed of the acoustic waves becomes more than 580 m/s at the bottom side of the F-region (~150 km), which is much higher than that in the troposphere (~300 m/s) at the location of 0° and 135° in geographic latitude and longitude at 04:00 UT on January 15, 2022.

## Code availability

The software used to read, analyze, and plot the data, part of the publicly available SPEDAS 5.0 software package including ERG plug-in tools (https://ergsc.isee.nagoya-u.ac.jp/analysis/spedas/index.shtml.en), is available at http://spedas.org/wiki/index.php?title=Downloads_and_Installation and can be used without any restriction. The publicly available IDL Coyote Graphics System software (http://www.idlcoyote.com/index.html) is used for creating two-dimensional maps of the data shown in Figs. 1, 4, and 6.
