# Supplementary material for: Generation of equatorial plasma bubble after the 2022 Tonga volcanic eruption
Source: Sci Rep. 2023 May 22;13:6450. doi: 10.1038/s41598-023-33603-3 (PMC10203289; doi:10.1038/s41598-023-33603-3)
Supplement: Supplementary file 2 — Supplementary Information. [file 41598_2023_33603_MOESM2_ESM.pdf]

SUPPLEMENTARY INFORMATION

Generation of equatorial plasma bubble after the 2022 Tonga volcanic eruption

Atsuki Shinbori, Takuya Sori, Yuichi Otsuka, Michi Nishioka, Septi Perwitasari, Takuo Tsuda, A. Kumamoto, Fuminori Tsuchiya, Shoya Matsuda, Yoshiya Kasahara, Ayako Matsuoka, Satoko Nakamura, Yoshizumi Miyoshi, Iku Shinohara

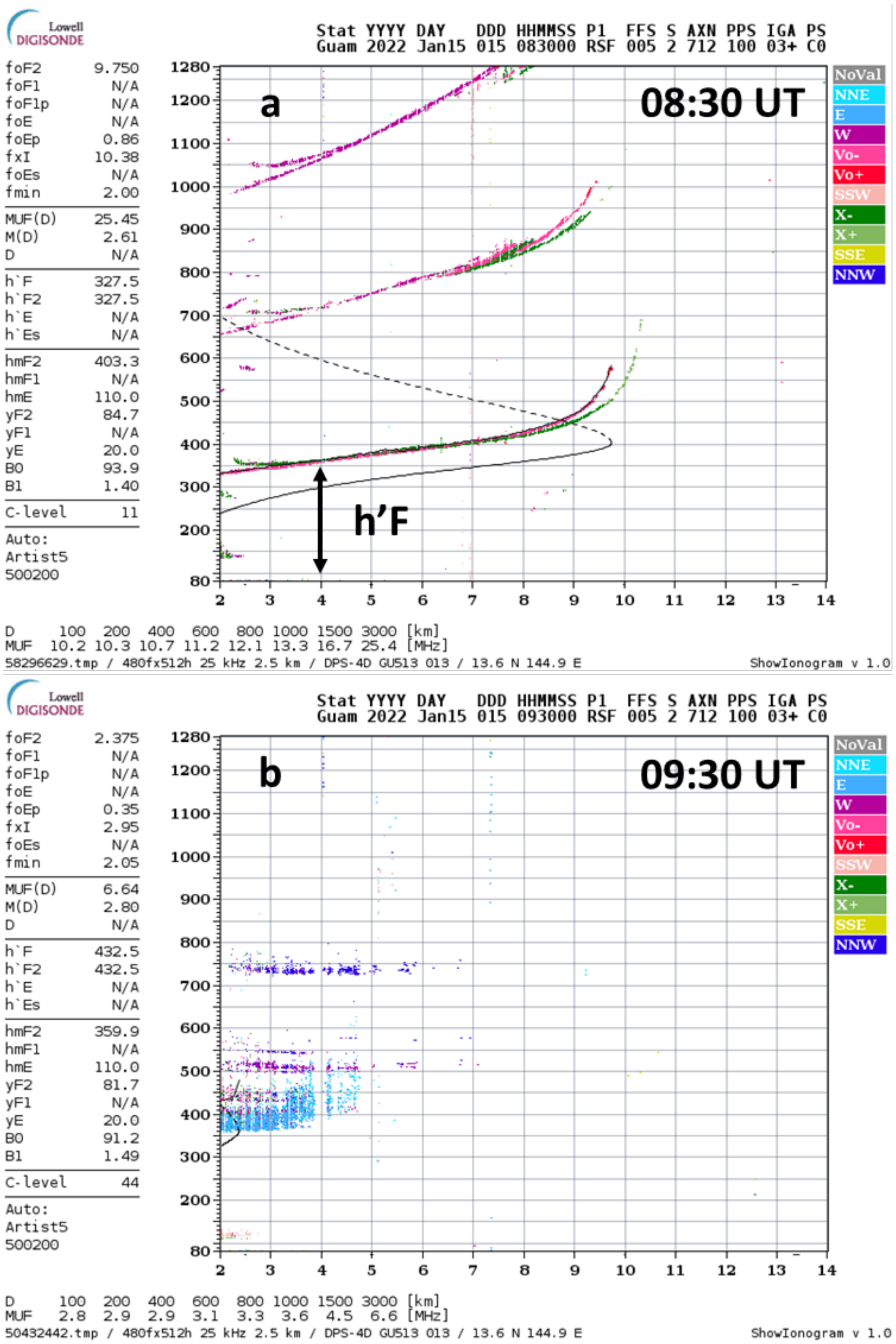

Fig. S1. Ionograms at 08:30 UT and 09:30 UT on January 15, 2022 as a function of frequency in MHz and virtual height in kilometers obtained from the ionosonde at the Guam station. These plots are open at the website (https://lgdc.uml.edu/common/DIDBYearListForStation?ursiCode=GU513).

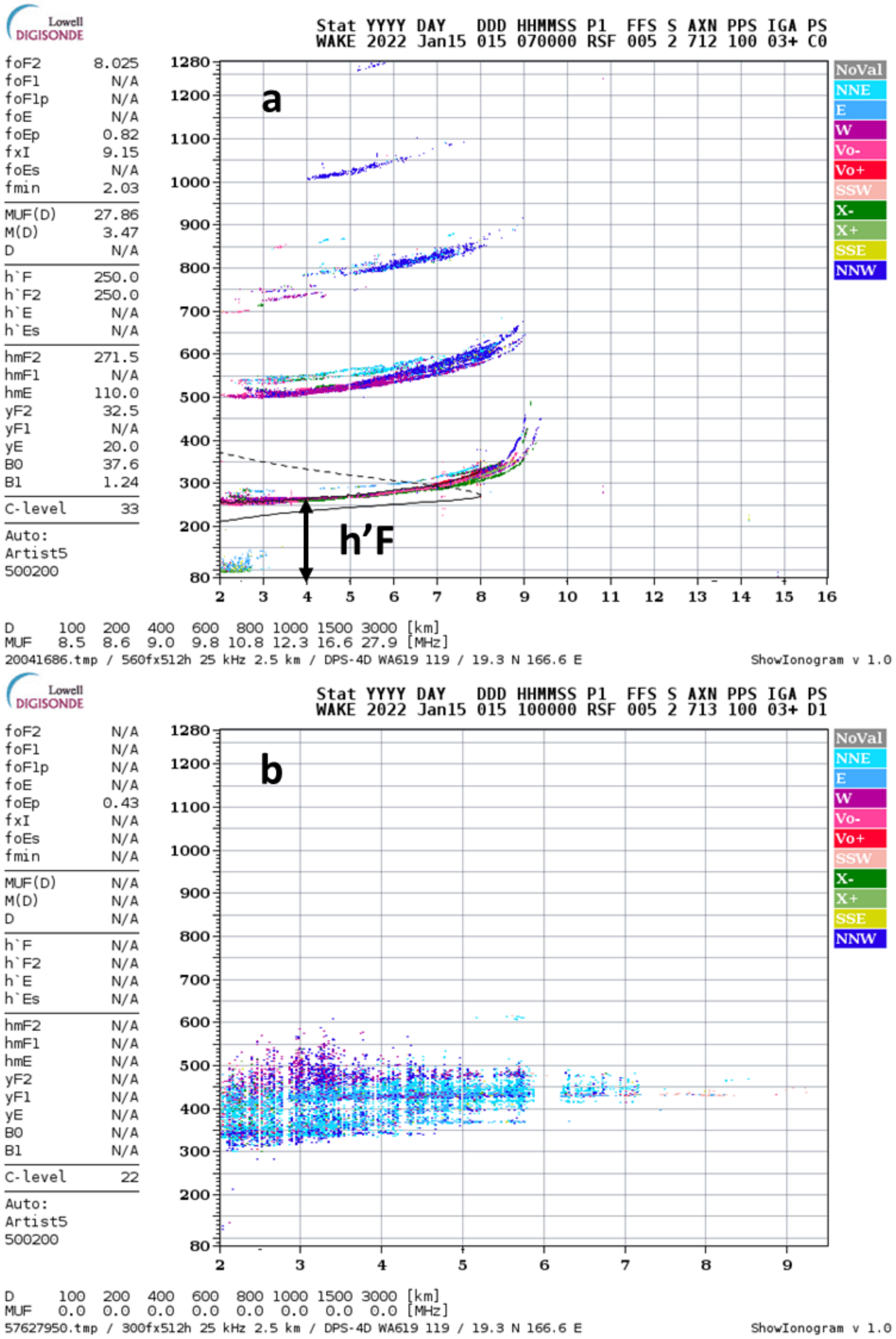

Fig. S2. Ionograms at 08:30 UT and 09:30 UT on January 15, 2022 as a function of frequency in MHz and virtual height in kilometers obtained from the ionosonde at the Wake station. These plots are provided in open access at the website (https://lgdc.uml.edu/common/DIDBYearListForStation?ursiCode=WA619).

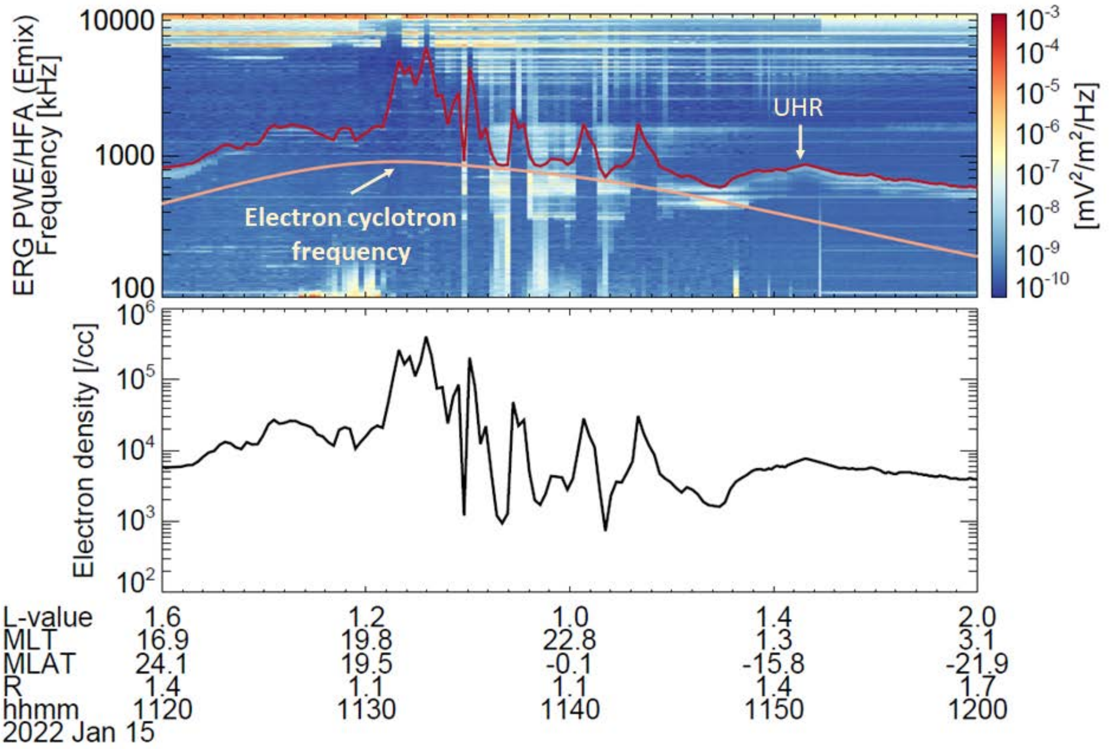

Fig. S3. Plasma wave dynamic spectra in a frequency range of 100–1000 kHz and electron density observed by the Arase satellite. The red and orange lines indicate the upper limit frequency of UHR waves and electron cyclotron frequency, respectively. The electron cyclotron frequency is determined by the measured magnetic field intensity. The parameters shown in the bottom panel represent the universal time and the location of the Arase satellite.

Movie S1.

This is the movie of the two-dimensional map of temperature deviation (d3) and ROTI as a function of geographic latitude and longitude during 04:00-13:50 UT on January 15, 2022. The time interval is 10 min. The ROTI and d3 values are marked by the color and grayscale codes, respectively. The horizonal dotted curves are the magnetic latitude on the ground calculated with the magnetic field model. The magnetic equator is represented by the red dotted curve. The blue and purple curves are the sunset terminators at a height of 105 and 300 km, respectively.
